# Visualizing veins from color images under varying illuminations for medical applications

**DOI:** 10.1117/1.JBO.26.9.096006

**Published:** 2021-09-20

**Authors:** Ru Jia, Chaoying Tang, Biao Wang

**Affiliations:** Nanjing University of Aeronautics and Astronautics, College of Automation Engineering, Nanjing, Jiangsu, China

**Keywords:** Monte Carlo simulation, optics, skin biophysics, blood, multiple regression analysis, multispectral image, RGB image, vein visualization

## Abstract

**Significance:** Effective vein visualization is critically important for several clinical procedures, such as venous blood sampling and intravenous injection. Existing technologies using infrared device or ultrasound rely on professional equipment and are not suitable for daily medical care. A regression-based vein visualization method is proposed.

**Aim:** We visualize veins from conventional RGB images to provide assistance in venipuncture procedures as well as clinical diagnosis of some venous insufficiency.

**Approach:** The RGB images taken by digital cameras are first transformed to spectral reflectance images using Wiener estimation. Multiple regression analysis is then applied to derive the relationship between spectral reflectance and the concentrations of pigments. Monte Carlo simulation is adopted to get prior information. Finally, vein patterns are visualized from the spatial distribution of pigments. To minimize the effect of illumination on skin color, light correction and shading removal operations are performed in advance.

**Results:** Experimental results from inner forearms of 60 subjects show the effectiveness of the regression-based method. Subjective and objective evaluations demonstrate that the clarity and completeness of vein patterns can be improved by light correction and shading removal.

**Conclusions:** Vein patterns can be successfully visualized from RGB images without any professional equipment. The proposed method can assist in venipuncture procedures. It also shows promising potential to be used in clinical diagnosis and treatment of some venous insufficiency.

## 1 Introduction

Venipuncture is one of the most common clinical procedures in everyday life. In general, it is used for venous blood sampling or intravenous injection. Hands and forearms are the main venipuncture sites. In clinical treatment, when trying to eliminate varicose veins and spider veins, clinicians also look for puncture sites to inject a sclerosant medication.^1^ At present, the most common way to locate veins is still to see with naked eyes or to touch with fingers, which depends significantly on the clinicians’ experience. For patients with thick fat, narrow veins, dark skin tone, or excessive body hair, the success rate of vein puncture may be decreased. Moreover, in some highly contagious disease contexts, such as COVID-19, the clinicians must wear medical goggles and surgical gloves, which makes the operation more difficult. Venipuncture failure would increase the suffering of patients both physically and psychologically. Therefore, an effective vein visualization device is needed. Existing technologies include infrared/near-infrared imaging,^1^^,^^2^ transillumination imaging,^3^ multispectral imaging,^4^^,^^5^ and ultrasound imaging.^6^ The first three technologies mainly use the difference in absorption properties between venous blood and other tissues to visualize veins, whereas ultrasound imaging utilizes soundwaves to detect vein structures and venous blood flow. However, the above technologies rely on professional equipment that is high cost and not suitable for daily medical care or telemedicine. Besides, some equipment needs direct skin contact, which cannot be used in patients with fragile skin and is not hygienic from a public health perspective. Therefore, it is critical to propose a simple, effective, and contactless vein visualization technology for daily medical treatment.

In this paper, we propose a regression-based method to visualize veins from color skin images taken by conventional digital cameras. No other professional equipment is required. We start with the analysis of light propagating in skin. Skin is a multilayered, inhomogeneous tissue. When light enters the skin, it is scattered, reflected, or absorbed. The reflected part is captured by human eyes or a camera to form the color we see. Based on this, we inverse the light–tissue interaction and color formation process to obtain skin properties from skin color. In this way, veins can be visualized from color skin images. We evaluate the proposed method on a dataset of 60 subjects and demonstrate that it can perform better than the state-of-art methods both qualitatively and quantitatively. The remainder of this paper is organized as follows. Section 2 briefly overviews related work in vein visualization. Section 3 discusses the proposed regression-based method. Section 4 reports the experimental results. Section 5 offers the conclusion.

## 2 Related Works

Recently, some technologies for visualizing veins from color skin images have been proposed. Tang et al.^7^ proposed a vein visualizing method based on image mapping. They extracted information from a pair of synchronized color and near-infrared images and used a neural network to map RGB values to NIR intensities. However, the model is completely learned from a dataset, so it is only a numerical solution. In addition, when the lighting condition changes, the model may get unreliable results. Tang et al.^8^ also proposed a vein visualization method based on optics and skin biophysics. They model skin color formation process based on Kubelka–Munk theory and then use a neural network to fit the inverse process. Vein patterns are derived from the distributions of the biophysical parameters from the inverse model. It does not rely on synchronized color and near-infrared images as the training set, but the inverse process is still based on the neural network approximation. In addition, all the deep learning-based vein visualization methods^9^^,^^10^ encounter the “black box” problem, which makes it difficult to improve the algorithm theoretically. Watanabe and Tanaka^11^ visualized veins by emphasizing the saturation of a color image. The algorithm is only based on image enhancement. For veins that are invisible in the color skin images, this method shows no result. Song et al.^12^ proposed a vein visualization method based on Weiner estimation using smart phone cameras. Reflectance images were reconstructed from conventional RGB images, and the 620-nm reflectance image was chosen to visualize veins. However, the reflectance images in 620 nm are not clear enough to show vein patterns because visible light cannot achieve the penetration depth as the near-infrared light. Thus, a postimage processing method was then employed to enhance contrast. Moreover, their method requires calibration for each camera device and illumination, which is not practical for widespread use. Sharma and Hefeeda^13^ also visualized veins from reconstructed spectral images. They used deep learning method to map RGB images to hyperspectral images in the range of 820 to 920 nm. Their method achieved good results, but training the model needs hyperspectral images, which are expensive to obtain.

## 3 Methodology

In an RGB image, veins are almost invisible to the naked eye because the pixels have very similar intensity values to those of other skin tissues. However, the biophysical parameters of veins and generic tissue are significantly different, which makes it possible to uncover vein patterns from their spatial distribution. This is the key idea of the regression-based method.

The color of the skin mainly depends on the skin structure and various pigments in the skin.^14^ Melanin and hemoglobin are the two main pigments. The properties of environment illumination and camera are also key factors in the process of color formation. Mathematically, the color formation process can be expressed as follows:^8^

$$[R,G,B]=f(E(\lambda ),S(\lambda ),C_{m},C_{b}),$$ (1)

where E(λ) represents the illuminant, S(λ) represents the spectral response functions of a camera. Here, $C_{m}$ and $C_{b}$ are the concentrations of melanin and blood, respectively. The color formation process is a well-posed problem, i.e., given the specific values of biophysical properties, illuminant, and camera model, the RGB values of a pixel can be uniquely determined. For example, Zoller and Kienle^15^ developed software that can generate the image of a blood vessel in skin according to specific input parameters. On the contrary, the inverse process $f^{-1}$ is an ill-posed problem, which is more complicated and can lead to multiple solutions. Therefore, *a priori* information should be imposed on the model to obtain the most possible solution.

The proposed regression-based method first preprocesses the input images to minimize illumination influence and remove shading effects. Diffuse reflectance spectral images are then reconstructed from the preprocessed images using human skin reflectance database as *a priori* information. Finally, the multiple regression analysis is applied to diffuse reflectance spectral images to derive the spatial distribution of melanin and blood based on Lambert–Beer law. Monte Carlo (MC) method is adopted in advance to simulate light propagating in skin to get the diffuse reflectance with varying skin parameters. The spatial distribution of blood can explicitly reflect vein patterns because veins contain much higher concentration of blood than other skin components.

### 3.1 Color Skin Image Preprocessing

#### 3.1.1 Light correction

As shown in Eq. (1), skin color is easily influenced by illumination variation in color formation processes. However, in the real world, the illumination conditions are usually unpredictable and uncontrolled,^16^ which will bring error to the later estimation of biophysical parameters in Sec. 3.3.2. Therefore, light correction is critical for widening the practical application of the vein visualization method.

In this section, an adaptive gamma correction method^17^ is applied on the color skin images. The main aim of the method is to calculate the best restoration $\gamma^{*}$ automatically to maximize the entropy of the transformed image, i.e., after correction, it can be assumed that the image contains most sufficient information. Unlike natural images that usually have rich color diversity, color images of skin have very similar RGB values, and color is the most important information in the biophysical parameters’ estimation process. Therefore, to attenuate the color distortion caused by uneven light, we calculate $\gamma^{*}$ from the gray-scale image and apply it to each channel of the RGB images, instead of performing corrections only on the V channel.

The best restoration $\gamma^{*}$ can be computed as^17^

$$\gamma^{*}=-\frac{1}{\frac{1}{N}\underset{m\in \Omega }{\sum }\ln(u_{m})},$$ (2)

where $u_{m}$ is the gray scale of the input image, Ω denotes the skin area of an image, and N is the valid number of pixels in Ω.

Then, the gamma correction is performed using $\gamma^{*}$ from Eq. (2) on each channel as

$$R^{\prime },G^{\prime },B^{\prime }=R^{\gamma^{*}},G^{\gamma^{*}},B^{\gamma^{*}}.$$ (3)

**Fig. 1:**
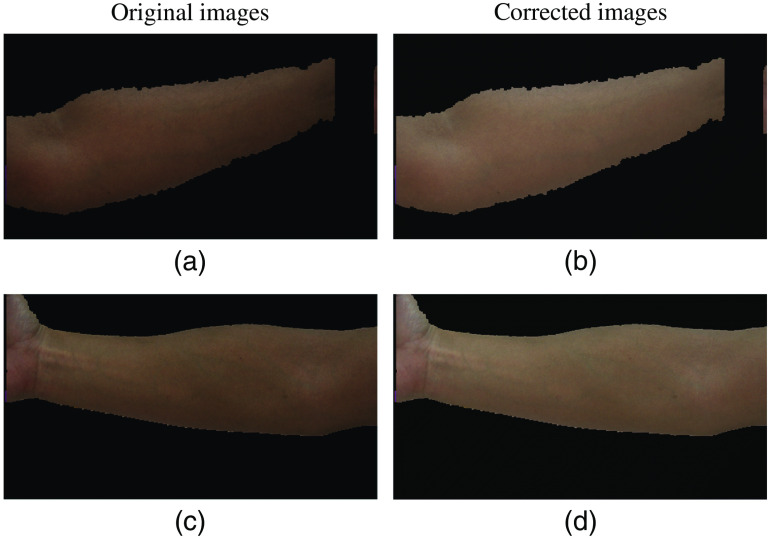
Color skin images before and after light correction. (a) and (c) Two color skin images; (b) and (d) corresponding light correction results with (b) γ*=0.694 and (d) γ*=0.758.

Figure 1 shows the original images and the images after light correction with corresponding $\gamma^{*}$ values. The uneven illumination condition is mitigated, and the skin color becomes more similar to their original state.

#### 3.1.2 Shading removal

Light correction only improves the holistic lighting conditions. However, arm skin has a curved surface. When illuminated by a directional light, the incident angle varies across the skin, which will result in shading effect. This section gives a detailed analysis on mechanism of the color formation process and then propose an algorithm to remove shade from skin.

The color of an RGB image is given as

$$I_{i}(x,y)=\int_{0}^{\infty }S_{i}(\lambda )E(\lambda )r(x,y,\lambda )w_{d}(x,y)d\lambda ,$$ (4)

where $I_{i}(x,y)(i=R,G,B)$ represents skin image intensity at pixel (x,y) after lighting correction, $S_{i}(\lambda )$ are the spectral response functions of a camera, E(λ) is the illuminant, and r(x,y,λ)represents the diffuse reflectance of skin at pixel (x,y) and wavelength λ. Some papers consider human skin as a specular + diffuse model (e.g., Ref. 18); however, most of the images in our dataset have no highlights on skin. Therefore, we consider our skin model as a complete diffuse surface (i.e., Lambertian surface) and only diffuse reflectance is considered in our study. $w_{d}$ represents the shading effect caused by curved surface. It equals to the dot product of the surface normal and the lighting direction and is independent of wavelength.

In computer graphics, it is assumed that the spectral response function can be characterized by a Dirac delta function $S_{i}(\lambda )=S(\lambda_{i})\delta (\lambda -\lambda_{i})$ with$\int_{0}^{+\infty }S_{i}(\lambda )d\lambda =S(\lambda_{i})$.^19^ Under this assumption, the integral representation Eq. (4) can be rewritten into a multiplicative form,

$$I_{i}(x,y)=S\;(\lambda_{i})E\;(\lambda_{i})r\;(x,y,\lambda_{i})w_{d}(x,y),$$ (5)

where $\lambda_{i}$ is the wavelength corresponding to the maximum value of spectral response function. Then, we take the logarithm of Eq. (5) to obtain the additive form. Before taking the logarithm, $I_{i}(x,y)$ should be scaled to [0,255] to avoid negative intensity:

$$\ln\;I_{i}(x,y)=\ln\;S(\lambda_{i})+\ln\;E(\lambda_{i})+\ln\;r(x,y,\lambda_{i})+\ln\;w_{d}(x,y).$$ (6)

It can be seen that only the last two terms are dependent on the position of image pixels. Between the two terms, shading $w_{d}(x,y)$ is usually a low-frequency variable that changes smoothly over the skin area, whereas reflectance $r(x,y,\lambda_{i})$ is a high-frequency variable because reflectance is dependent on the concentration of pigments that distributes inhomogeneously in the skin.^18^ A bilateral filter is a nonlinear filter that can preserve edges and reduce noises in images. In this study, it was adopted iteratively such that the high-frequency reflectance will gradually be smoothed out and the low-frequency illumination and shading effects will remain. The performance of the bilateral filter relies on the value of the spatial standard deviation $\sigma_{1}$ and intensity standard deviation $\sigma_{2}$. Inspired by Ref. 18, in our experiment, we choose $\sigma_{1}$ and $\sigma_{2}$ as

$$\sigma_{1}=0.05*\min(R_{x},R_{y}),$$ (7)

$$\sigma_{2}=0.05*\max(I_{\mathrm{remain}}),$$ (8)

where $R_{x}$ and $R_{y}$ are the width and height of the image, respectively. $I_{\mathrm{remain}}$ is the input image of the bilateral filter in each iteration. 0.05 was chosen for both the spatial coefficient and intensity coefficient. Experiment on our 60 subjects indicates that this combination can achieve the best decomposition results.

After bilateral filtering, the low-frequency component is defined as $\ln(I_{\mathrm{base},i})$ and the high-frequency component is defined as $\ln(I_{\mathrm{detail},i})$ with $\ln(I_{\mathrm{base},i})+\ln(I_{\mathrm{detail},i})=\ln(I_{i})$. It should be noted that vein pattern information will finally be extracted from pigment distribution, so it is embodied in the detail image. Therefore, to eliminate the shading effect, we keep the detail layer and add the global mean of illuminant layer as a base color to obtain the corrected image, i.e.,

$$\ln(I_{\mathrm{correct},i}(x,y))=\ln(I_{\text{detail},i}(x,y))+\frac{1}{N}\underset{(x,y)\in \Omega }{\sum }\ln(I_{\text{base},i}(x,y))(i=R,G,B).$$ (9)

**Fig. 2:**
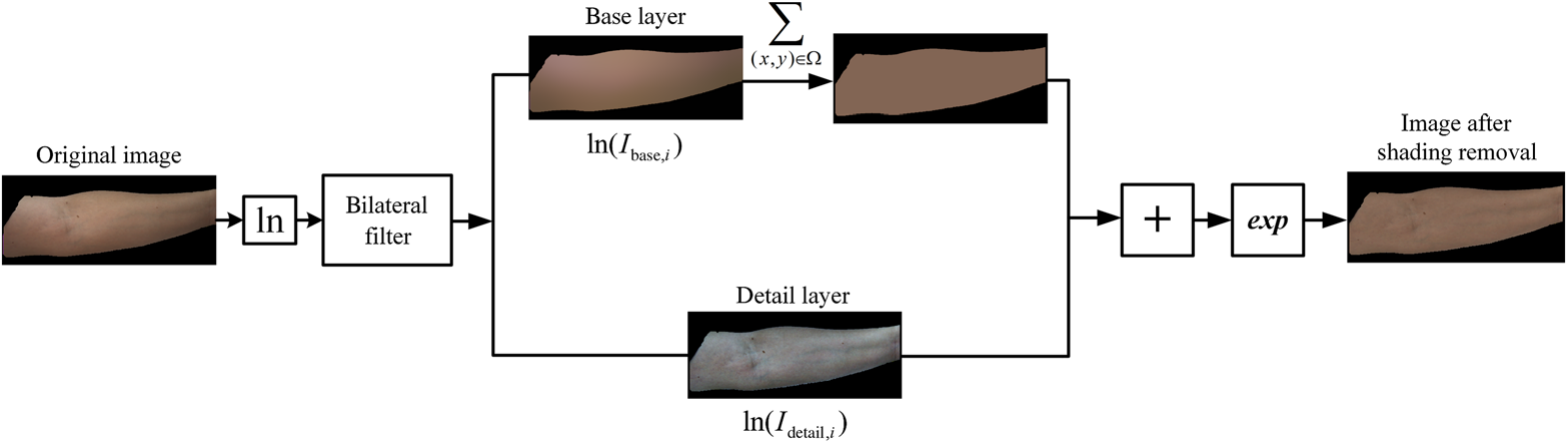
Shading removal process.

The process of shading removal is shown in Fig. 2.

### 3.2 Spectral Image Reconstruction

In this section, Wiener estimation was performed to reconstruct the spectral reflectance images from an RGB image. After shading removal, Eq. (4) can be rewritten as

$$I_{i}(x,y)={\overset{¯}{\omega }}_{d}\int_{\;0}^{\;\infty }S_{i}(\lambda )E(\lambda )r(x,y,\lambda )d\lambda (i=R,G,B),$$ (10)

where ${\bar{\omega }}_{d}$ is the constant coefficient and can be fitted into the illuminant term. Equation (10) is then discretized in the wavelength range of 400 to 700 nm at an interval of 10 nm and rewritten into vector notation as

I=SEr, (11)

where S is a 3×31 matrix and each row represents camera spectral response function of each channel. E is a 31×31 diagonal matrix and represents the spectrum of illuminant. r is a 31×1 vector representing the reflectance spectrum of a pixel in an image. $I=[R,G,B{]}^{T}$ is the corresponding color of the pixel.

The Wiener estimation of r is given as

$$\overset{˜}{r}=\mathrm{WI}.$$ (12)

The Winer estimation matrix W is calculated by minimizing the square error $\left\langle |r-\overset{˜}{r}|\right\rangle$, where ⟨·⟩ denotes the ensemble average. W is derived as^20^

$$W=\langle {\mathrm{rI}}^{T}\rangle \langle {\mathrm{II}}^{T}\rangle^{-1}=\langle {\mathrm{rr}}^{T}\rangle F^{T}(F\langle {\mathrm{rr}}^{T}\rangle F^{T}{)}^{-1},$$ (13)

where F=SE. We assume that S and E are known. In this study, D65 illuminant and JAI AD-080-GE camera are chosen. ${\mathrm{rr}}^{T}$ is the autocorrelation matrix which should be obtained from prior knowledge. In this study, we use a skin reflectance database^21^ consisting of 4392 spectral reflectance as prior knowledge. The database consists of nine body areas of 482 subjects from three ethnic groups, which are Caucasian, Chinese, and Kurdish. The reflectance is measured using a Minolta CM-2600d spectrophotometer. We extract reflectance in the range of 400 to 700 nm and calculate the average ${\mathrm{rr}}^{T}$for the 4392 subjects to get $\left\langle {\mathrm{rr}}^{T}\right\rangle$ in Eq. (13).

**Fig. 3:**
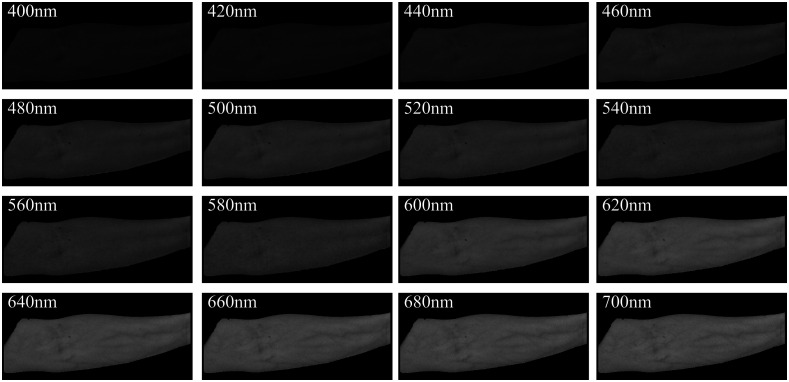
Spectral reflectance images in the range of 400 to 700 nm at an interval of 20 nm.

**Fig. 4:**
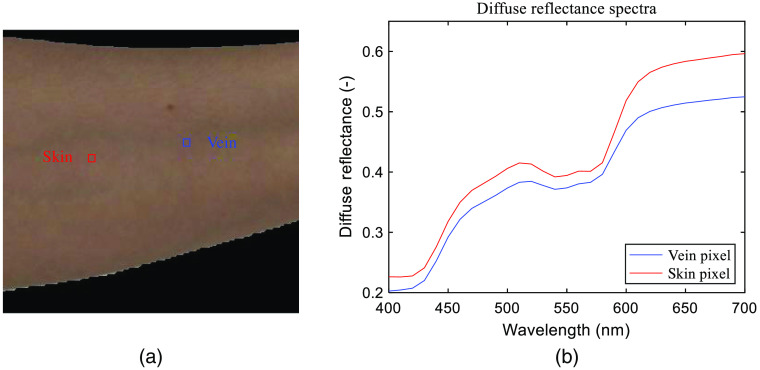
Reconstructed spectral reflectance of two pixels. (a) A skin area marked with a skin pixel (red square) and a vein pixel (blue square); (b) their diffuse reflectance spectra reconstructed from Wiener estimation.

Finally, Eq. (12) is performed to each pixel of the preprocessed arm skin image to get the spectral reflectance images. The result is shown in Fig. 3. The reconstructed diffuse reflectance spectra of two pixels are shown in Fig. 4. It can be seen that their shapes are consistent with the diffuse reflectance spectra of human skin. The diffuse reflectance of vein is lower than that of skin because blood absorbs more light than generic skin, especially in the red wavelength range.

### 3.3 Vein Patterns Visualization

In this section, the vein patterns are finally visualized from the distribution of blood. As it is mentioned earlier, estimating biophysical parameters from the spectral reflectance images is an ill-posed problem, i.e., the analytical model is difficult to find, and there are many possible solutions. In this section, MC simulation is used as the forward model to construct a biophysical parameters-spectral reflectance dataset as prior information. Then, the relationship between the absorbance spectrum and pigment concentrations is derived from the dataset based on Lambert–Beer law.

#### 3.3.1 Forward model for light transport in skin

Before MC simulation, we formulate a general model to describe the skin structure and define each layer’s optical properties. In this study, we model skin as a three-layer structure, which are epidermis, dermis, and hypodermis. The optical properties required in MC simulation are absorption coefficient $\mu_{\alpha }(\lambda )\;({\mathrm{cm}}^{-1})$, scattering coefficient $\mu_{s}(\lambda )\;({\mathrm{cm}}^{-1})$, anisotropy factor g(λ), refractive index n, and thickness d(cm).

The absorption coefficients $\mu_{\alpha }(\lambda )$ of the epidermis and the dermis are mainly dependent on the concentrations of melanin in epidermis and hemoglobin in dermis. We choose $\mu_{\alpha }(\lambda )$ published by Donner and Jensen^22^ for epidermis and dermis, and $\mu_{\alpha }(\lambda )$ published by Atencio et al.^23^ for hypodermis. The optical properties in Ref. 22 are for human skin and those in Ref. 23 are for neonatal forehead skin. The values of $\mu_{s}(\lambda )$ are also chosen from Refs. 22 and 23. The anisotropy factor g(λ) is chosen from Ref. 24 for epidermis and dermis and Ref. 23 for hypodermis. The refractive index n is set to be 1.37 for epidermis and dermis and 1.44 for hypodermis.^23^ The thickness values d are set to be 0.006,^25^ 0.09,^23^ 0.03 cm,^23^ respectively. The optical properties in Refs. 24 and 25 are also for human skin.

**Fig. 5:**
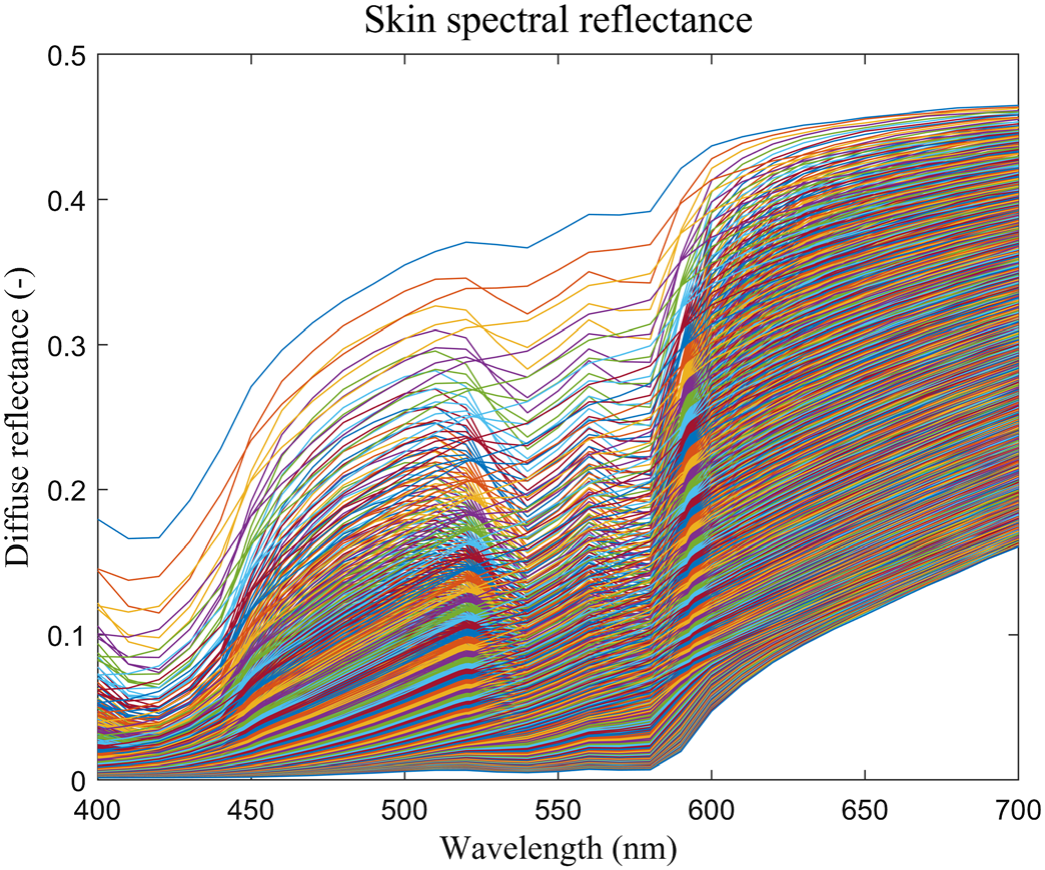
2500 spectral reflectance obtained from MC simulations.

To cover the color variation of skin as diverse as possible, reasonably wide ranges of $C_{m}$ and $C_{b}$ are chosen, i.e., $C_{m}$ from 1.3% to 43%^26^ and $C_{b}$ from 0.1% to 7%. Both of the ranges are uniformly divided into 50 points and consequently result in 2500 $\left(C_{m},C_{b})∼r(\lambda \right)$ data pairs. The simulated skin spectral reflectance is shown in Fig. 5. In this study, we utilize a GPU-accelerated MC simulation tool CUDAMCML,^27^ which can accelerate the simulations by about three orders of magnitude than running sequentially on a CPU. The calculation time for one spectrum from 400 to 700 nm at the interval of 10 nm is ∼11 s, using NVIDIA GeForce GT 710 card.

#### 3.3.2 Inverse model based on multiple regression analysis

Using the forward model and the established dataset in Sec. 3.3.1, we try to find the inverse model in this section. First, the diffuse reflectance spectrum r(λ) is transformed into the absorbance spectrum A(λ) by^28^

$$A(\lambda )=-\log_{10}(r(\lambda )).$$ (14)

According to the modified Lambert–Beer law, the absorbance spectrum A(λ) can be expressed as

$$A(\lambda )=C_{m}l_{\mathrm{epi}}\varepsilon_{m}(\lambda )+C_{\mathrm{ob}}l_{\mathrm{der}}\varepsilon_{\mathrm{ob}}(\lambda )+C_{\mathrm{db}}l_{\mathrm{der}}\varepsilon_{\mathrm{db}}(\lambda )+D(\lambda ),$$ (15)

where $C_{m},C_{\mathrm{ob}},C_{\mathrm{db}}$ are the molar concentrations of melanin, oxygenated blood, and deoxygenated blood, respectively, and $C_{b}=C_{\mathrm{ob}}+C_{\mathrm{db}}$. $l_{\mathrm{epi}}$ and $l_{\mathrm{der}}$ denote the mean path length in epidermis and dermis, respectively. ε(λ) denotes the molar extinction coefficients of three pigments.^29^ D(λ) indicates the absorbance of other minor components and scattering loss.

Second, we regard absorbance spectrum as response variable and extinction coefficients as predictor variables and then transform Eq. (15) into a multiple regression model,^28^

$$A(\lambda )=a_{m}\varepsilon_{m}(\lambda )+a_{\mathrm{ob}}\varepsilon_{\mathrm{ob}}(\lambda )+a_{\mathrm{db}}\varepsilon_{\mathrm{db}}(\lambda )+a_{0},$$ (16)

where $a_{m},a_{\mathrm{ob}},\text{and }a_{\mathrm{db}}$ are the regression coefficients describing the contributions of each ε(λ) to A(λ), and are closely related to $C_{m}$, $C_{\mathrm{ob}}$, and $C_{\mathrm{db}}$, respectively.^28^ However, $a_{m}$ is not only dependent on $C_{m}$ but also influenced by $C_{\mathrm{ob}}$ and $C_{\mathrm{db}}$ because although the mean path length in epidermis $l_{\mathrm{epi}}$ is mainly determined by melanin in epidermis, it is also affected by pigments in the dermis due to the complexity of light–tissue interaction. This conclusion also applies to the $a_{\mathrm{ob}}$ and $a_{\mathrm{db}}$. It indicates that $a_{m},a_{\mathrm{ob}},a_{\mathrm{db}},a_{0}$ and $C_{m},C_{\mathrm{ob}},C_{\mathrm{db}}$ are interdependent.^28^ So, another multiple regression model is used to establish the relationship between $C_{m},C_{b}$ and $a_{m},a_{\mathrm{tb}},a_{0}$:

$$C_{m}=a\cdot b_{m},$$ (17)

$$C_{b}=a\cdot b_{\mathrm{tb}},$$ (18)

$$a=[1,a_{m},a_{\mathrm{tb}},a_{0},a_{m}^{3},a_{\mathrm{tb}}^{3},a_{0}^{3},a_{m}\cdot a_{\mathrm{tb}}\cdot a_{0},a_{m}^{2}\cdot a_{\mathrm{tb}},\;a_{m}^{2}\cdot a_{0},a_{\mathrm{tb}}^{2}\cdot a_{m},a_{\mathrm{tb}}^{2}\cdot a_{0},a_{0}^{2}\cdot a_{m},a_{0}^{2}\cdot a_{\mathrm{tb}}],$$ (19)

where $a_{\mathrm{tb}}=a_{\mathrm{ob}}+a_{\mathrm{db}}$, a is a 1×14 vector containing $a_{m},a_{\mathrm{tb}},a_{0}$ and their third order terms. $b_{m}$ and $b_{\mathrm{tb}}$ are 1×14 vectors that should be derived in advance based on the dataset from MC using Eqs. (1619). It should be noted that multiple regression analysis is only performed in 500 to 600 nm at interval of 10 nm rather than in 400 to 700 nm because the spectral features of oxyhemoglobin and deoxyhemoglobin differ more significantly in this range than in the whole visible range and thus can lead to a better separation. For the 2500-absorbance spectra derived from MC simulations, the mean value of the $R^{2}$ statistic is 0.977±0.010 in 500 to 600 nm whereas it is only 0.905±0.069 in 400 to 700 nm.

Once $b_{m}$ and $b_{\mathrm{tb}}$ are obtained, we can perform multiple regression analysis Eq. (16) on each pixel of spectral reflectance images in 500 to 600 nm at the interval of 20 nm and get vector a for each pixel. Later, we can use Eqs. (17) and (18) to get the spatial distribution of $C_{m}$ and $C_{b}$, where vein patterns can be observed.

## 4 Experimental Results

To evaluate the proposed regression-based method, we collected synchronized RGB/NIR images of inner arms from 60 subjects. The subjects are all Chinese. There were not protocols or eligibility criterion to recruit subjects. We invited as many subjects as possible to construct our skin image database. A JAI AD-080-GE industrial camera was used to capture images. JAI AD-080-GE camera is a 2-CCD camera providing simultaneous RGB/NIR images. When light enters the lens, it is separated by a prism into the visible/color part of the spectrum (400 to 700 nm) and the near-infrared part of the spectrum (750 to 1000 nm). A day light source and an NIR light source were used to illuminate the skin. During collection, one RGB image and one NIR image were captured simultaneously from inner arm of each subject. The RGB images are the test images and the NIR images are ground truth for comparison. D65 is the most commonly used daylight illuminant and is used in this paper as an illuminant. After collection, the skin area was segmented from the original images based on color. In the remaining section, we first validate the regression-based method on our dataset. Then, we evaluate the effect of light correction and shading removal on the regression-based vein visualization method. Three state-of-art methods are used for comparison, including: Watanabe’s image enhancement method,^11^ Song’s Wiener estimation method,^12^ and Tang’s optical method.^8^ Finally, we test the regression-based method on the spider vein images.

**Fig. 6:**
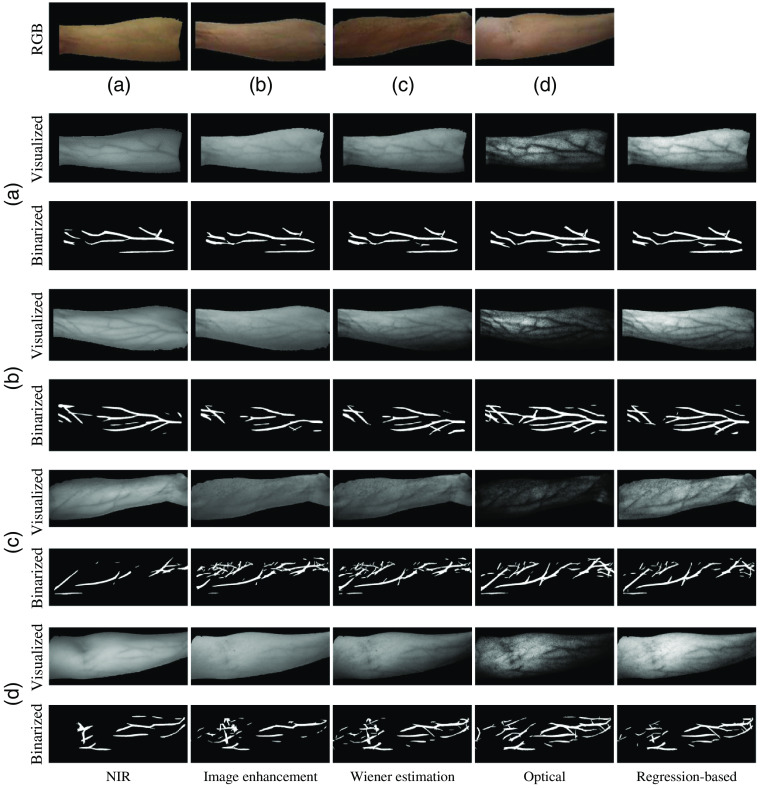
Vein visualization and extraction results.

To objectively evaluate the proposed method, we extracted vein patterns from both the NIR images and visualized images and compared them pixel-by-pixel. We used the same extraction process as stated in Refs. 10 and 30. At first, we used a filter bank composed of the real parts of 16 Gabor filters with different scales and orientations to get the location of veins. Then, the information images of veins were enhanced and binarized to get the final vein patterns. In the extracted vein patterns, the vein pixels were labeled as 1 and background pixels as 0, which is shown in Fig. 6. Using the vein patterns extracted from NIR images as ground truth, four metrics were calculated to measure the algorithm’s performance, which are accuracy, precision, recall, and F1 score. Mathematically, they are expressed as

$$\mathrm{Accuracy}=\frac{\mathrm{TP}+\mathrm{TN}}{\mathrm{TP}+\mathrm{TN}+\mathrm{FP}+\mathrm{FN}},$$ (20)

$$\mathrm{Precision}=\frac{\mathrm{TP}}{\mathrm{TP}+\mathrm{FP}},$$ (21)

$$\mathrm{Recall}=\frac{\mathrm{TP}}{\mathrm{TP}+\mathrm{FN}},$$ (22)

$$F_{1}=\frac{2*\mathrm{Precision}*\mathrm{Recall}}{\mathrm{Precision}+\mathrm{Recall}}.$$ (23)

The confusion matrix in our case is defined as given in Table 1.

### 4.1 Validation of the Vein Visualization Method

Figure 6 shows four examples of the experimental results. In Fig. 6, the first row shows four color skin images from our dataset. It should be noted here that the skin boundaries are not exactly smooth, because some background pixels near the boundary have similar value as the skin pixels, so they may be easily misclassified as skin during segment. However, the rough boundary has no impact on vein visualization results, because veins gather in the middle part of arms. The second and third rows show the vein visualization and extraction results from Fig. 6(a). The second row shows the corresponding NIR image, the visualization results from the image enhancement method, the Wiener estimation method, the optical method, and the regression-based method, respectively. The third row shows the corresponding vein patterns extracted from the second row. The remainder of Fig. 6 is the vein visualization and extraction results from Figs. 6(b)–6(d). The objective evaluation of the four examples and the means of all 60 images are given in Table 2. The image enhancement method is simplest among the four methods, and it visualizes veins only by emphasizing saturation of the whole image and then extracting R channel. Therefore, the visualized results from their method usually contain less noise, which consequently leads to higher precision, since noises in the visualized images are easily mistaken as small veins in the extracted vein patterns. On the other hand, the vein lines from the image enhancement method are relative dim, making the extracted vein patterns less complete and lead to lower recall. [e.g., in Fig. 6(b), the binarized vein patterns in the second column are less complete than those in the first column.] For hairy skin [e.g., Fig. 6(c)], the image enhancement method’s performance is poor. The recall of the optical method is the highest in all the examples, because the vein patterns obtained from their method are the most distinct. However, the noises are also heavier in these images, resulting in low precision. The Wiener estimation method and the proposed method have a better trade-off between precision and recall. For the 60-arm images in our dataset, the Wiener estimation method performs better than the proposed method. Therefore, in the next section, we apply light correction and shading removal on the original skin images to improve the performance.

**Table 1:** Confusion matrix.

		Vein patterns extracted from visualized images (Predicted)	
		Vein	Background
Vein patterns extracted from NIR images (ground truth)	Vein	TP	FN
	Background	FP	TN

**Table 2:** Objective evaluations of vein visualization methods.

Images	Metrics	Image enhancement	Wiener estimation	Optical	Regression-based
Fig. 6(a)	Accuracy	0.9920	**0.9928**	0.9916	0.9925
	Precision	**0.7859**	0.7830	0.7056	0.7478
	Recall	0.7798	0.8423	**0.9345**	0.8939
	F1 score	0.7828	0.8116	0.8041	**0.8143**
Fig. 6(b)	Accuracy	0.9874	0.9874	0.9850	**0.9876**
	Precision	**0.7640**	0.7170	0.6034	0.6819
	Recall	0.5881	0.6769	**0.8670**	0.7802
	F1 score	0.6646	0.6963	0.7116	**0.7277**
Fig. 6(c)	Accuracy	0.9665	0.9707	0.9711	**0.9751**
	Precision	0.2881	0.3377	0.3554	**0.3895**
	Recall	0.5947	0.6651	**0.7644**	0.6951
	F1 score	0.3881	0.4480	0.4852	**0.4992**
Fig. 6(d)	Accuracy	**0.9802**	0.9774	0.9723	0.9799
	Precision	**0.5594**	0.5035	0.4385	0.5460
	Recall	0.6189	0.7264	**0.7628**	0.7014
	F1 score	0.5877	0.5947	0.5569	**0.6140**
60 images	Accuracy	0.9796	**0.9797**	0.9778	0.9765
	Precision	0.4499	**0.4645**	0.4307	0.4099
	Recall	0.4723	0.5313	**0.7102**	0.5954
	F1 score	0.4502	0.4835	**0.5291**	0.4752

Note: The highest mean values are shown in bold.

**Fig. 7:**
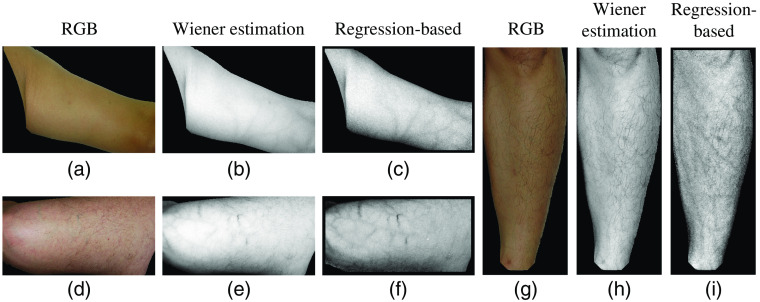
Vein visualization results in other skin areas. (a) The skin image of a left upper arm; (d) the skin image of a thigh; (g) the skin image of a front calf; (b) (e), and (h) the visualization results of the Wiener estimation method; and (c), (f), and (i) the visualization results of the regression-based method.

Since Wiener estimation is used in both the regression-based method and the Wiener estimation method, to further validate the effectiveness of the regression-based method, we compared the results of the two methods in other skin areas. Figure 7(a) shows the skin image of a left upper arm. Figure 7(d) shows the skin image of a thigh and there is a highlight above the knee. These areas contain more fat. Figure 7(g) shows the skin image of a front calf which contains more muscles. Figures 7(b), 7(e), and 7(h) show the results from the Wiener estimation method. Figures 7(c), 7(f), and 7(i) show the results from the regression-based method. The results from the Wiener estimation method can hardly show veins or cannot show veins at all, whereas the regression-based method can produce better visualization results. What is more, the regression-based method is less sensitive to light variation compared with the Wiener estimation method. That is because the Wiener estimation method only uses specific wavelength reflectance images, whereas the regression-based method is based on an accurate optical model. Therefore, the proposed method is robust to different parts of skin.

### 4.2 Evaluation of the Light Correction and Shading Removal Algorithms

**Fig. 8:**
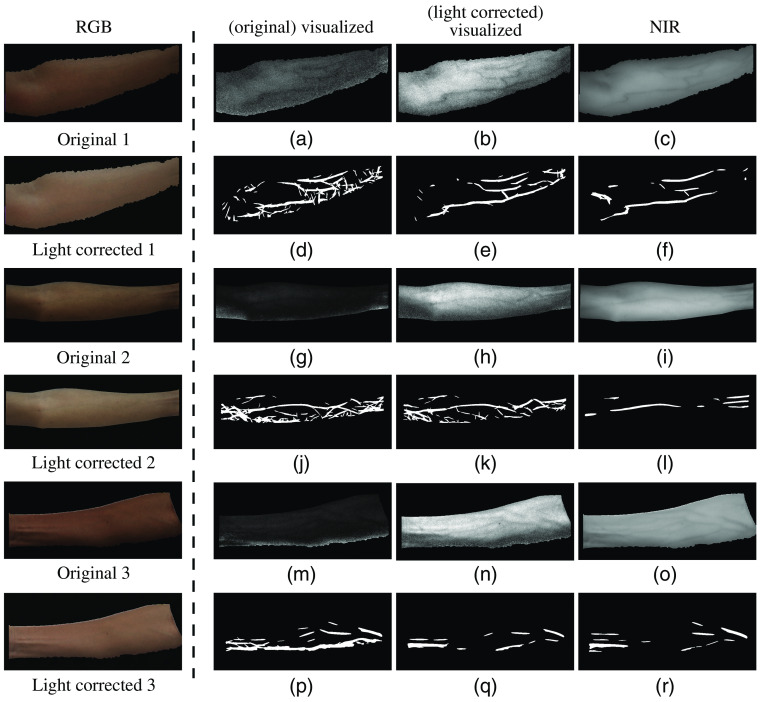
Vein visualization results before and after light correction. (a) The visualized result of original image 1; (b) the visualized result of light corrected image 1; (c) the corresponding NIR image; (d)–(f) the extracted vein patterns from (a)–(c), respectively. (g)–(l) and (m)–(r) Two sets of results for examples 2 and 3, respectively.

In this section, we evaluate the effect of light correction and shading removal on the performance of the regression-based vein visualization method. First, we compare the vein visualization results before and after light correction. Figure 8 shows some experimental results. The first column of Fig. 8 shows three sets of original skin images and the images after light correction. The second and third columns are the visualized results of the original skin images and the light corrected images, respectively, with their extracted vein patterns in the row below. The fourth column is the corresponding NIR images and their extracted vein patterns. The objective evaluation of the three examples and the means of all 60 images are shown in Table 3. It can be seen in Fig. 8 that in some skin areas, vein patterns are failed to be visualized from the original image due to the poor lighting condition, whereas from the corrected images, they are clearer. From the extracted vein patterns, we can see that the noises have been greatly reduced. For example, in Fig. 8(d), vein patterns are blurred with noises. After light correction, the noises have been greatly reduced and vein patterns become clearer and more complete as shown in Fig. 8(e). The objective evaluation also proves that the clarity and completeness of vein patterns are improved by light correction.

**Table 3:** Objective evaluation of light correction process.

Images	Accuracy	Precision	Recall	F1 score
Original 1	0.9711	0.3329	0.6527	0.4409
Light corrected 1	**0.9845**	**0.5425**	**0.7093**	**0.6148**
Original 2	0.9613	0.1673	0.6054	0.2622
Light corrected 2	**0.9720**	**0.2293**	**0.6208**	**0.3349**
Original 3	0.9793	0.1896	0.4327	0.2636
Light corrected 3	**0.9931**	**0.6011**	**0.5753**	**0.5879**
60 original images	0.9765	0.4099	0.5954	0.4752
60 light corrected images	**0.9791**	**0.4498**	**0.6178**	**0.5136**

Note: The highest mean values are shown in bold.

**Fig. 9:**
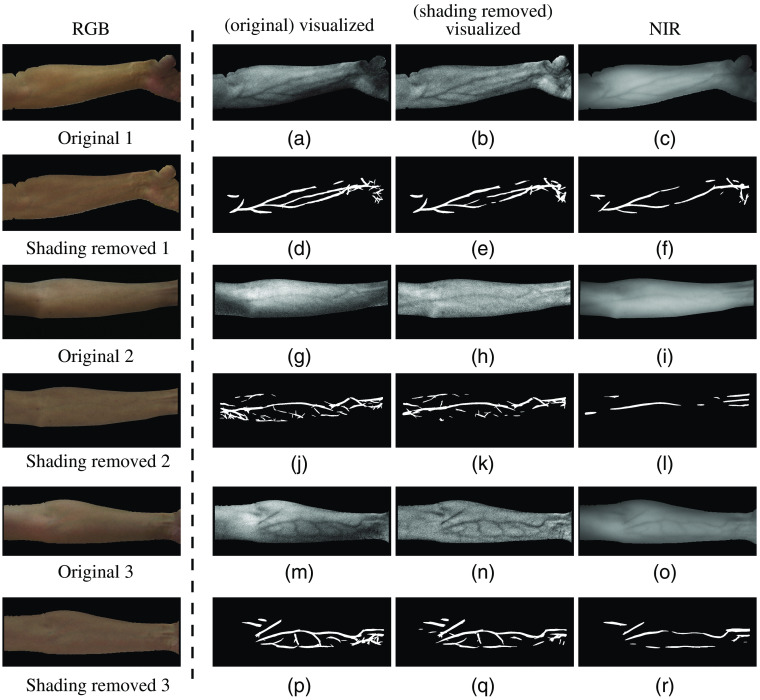
Vein visualization results before and after shading removal. (a) The visualized result of original image 1 (the image is after light correction); (b) the visualized result of shading removed image 1; (c) the corresponding NIR image; (d)–(f) the extracted vein patterns from (a)–(c), respectively. (g)–(l) and (m)–(r) Two sets of results for examples 2 and 3, respectively.

Second, we compare the vein visualization results before and after shading removal. Figure 9 shows some experimental results. The first column of Fig. 9 shows three sets of original skin images (after light correction) and their shading removed results. The second and third columns show the visualized results of the original skin images and the shading removed images, respectively, with their extracted vein patterns in the row below. The fourth column shows the corresponding NIR images and their extracted vein patterns. The objective evaluation of the three examples is shown in Table 4. It can be seen in Fig. 9 that after shading removal of the skin images, the intensities of generic skin become more uniform, making the veins more distinct from skin. Moreover, the visualized results from the shadow area of skin often contain a lot of noises, whereas in the shading removed images, the noises are reduced and vein patterns are clearer [e.g., the bottom left part of skin in Figs. 9(g) and 9(h), the skin near the lower boundary of arm in Figs. 9(m) and 9(n)]. The four objective evaluation metrics are also improved by shading removal process.

**Table 4:** Objective evaluation of shading removal process.

Images	Accuracy	Precision	Recall	F1 score
Original 1	0.9825	0.5555	0.7833	0.6500
Light corrected 1	**0.9855**	**0.6093**	**0.8394**	**0.7061**
Original 2	0.9720	0.2293	0.6208	0.3349
Light corrected 2	**0.9788**	**0.3092**	**0.7060**	**0.4301**
Original 3	0.9793	0.4641	0.8041	0.5885
Light corrected 3	**0.9831**	**0.5270**	**0.8227**	**0.6424**
60 original images	0.9791	0.4498	0.6178	0.5136
60 shading removed images	**0.9803**	**0.4718**	**0.6532**	**0.5414**

Note: The highest mean values are shown in bold.

**Fig. 10:**
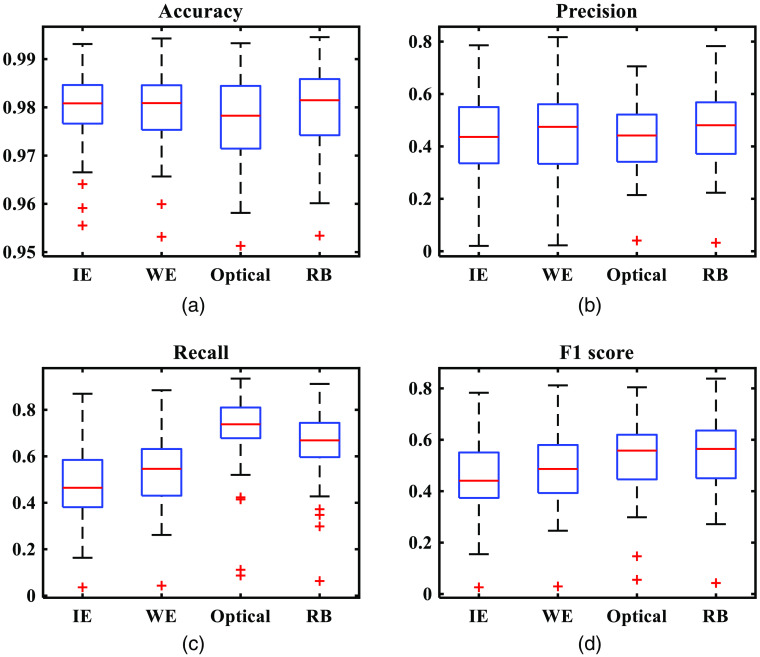
Boxplots of (a) accuracy, (b) precision, (c) recall, and (d) F1 score of the state-of-art methods and the regression-based method (60 images). It should be noted that IE stands for the image enhancement method, WE stands for the Wiener estimation method, and RB stands for the regression-based method.

To get the objective evaluation of the whole algorithm, the mean values and boxplots of the four metrics are shown in Table 5 and Fig. 10. It should be noted that the results compared now are obtained using the whole algorithm (including the vein visualization process, the light correction process, and the shading removal process). The results show that the regression-based method has the highest accuracy, precision, and F1 score. However, the recall is lower than that of the optical method. It indicates that further study is required to enhance vein patterns and reduce noise.

**Table 5:** Objective evaluation of the whole algorithm (60 images).

Methods	Accuracy	Precision	Recall	F1 score
Image enhancement	0.9796	0.4499	0.4723	0.4502
Wiener estimation	0.9797	0.4645	0.5313	0.4835
Optical	0.9778	0.4307	**0.7102**	0.5291
Regression-based	**0.9803**	**0.4718**	0.6532	**0.5414**

Note: The highest mean values are shown in bold.

### 4.3 Application for Spider Vein Treatment

Spider veins are small, damaged veins that appear on the surface of legs or face. They are usually caused by valves inside some veins having weakened or damaged.^31^ These veins are named as “feeder veins.” When the valves inside the feeder veins stop working normally, blood may pool inside the veins and cause continuous venous hypertension that makes capillaries connected to the feeder veins become enlarged and bulge. In legs, spider veins are usually the early symptom of varicose veins. The treatments for spider veins include sclerotherapy, phlebectomy, and laser treatment. Sclerotherapy involves injecting a medicine called sclerosant to the affected veins, making them to shrink. Phlebectomy is a minimally invasive surgery to remove some large, damaged veins, while laser treatment is a noninvasive procedure that uses a focused beam of light to destroy smaller veins. All the treatments need to find the affected veins first, and they all need to combine the treatment of the feeder veins with that of the spider veins. Otherwise, spider veins may reappear after some time although they disappear initially.^32^

**Fig. 11:**
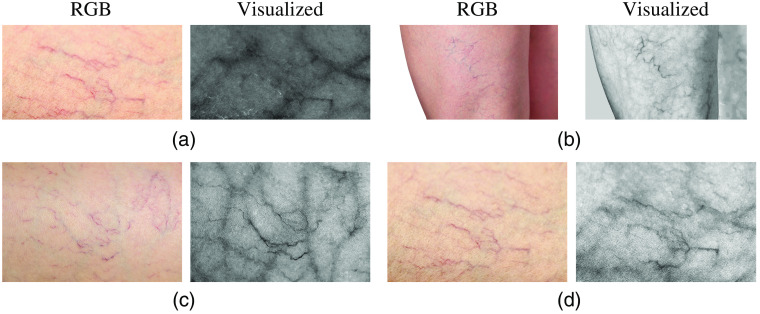
Vein visualization results on spider vein images. (a)–(d) Four pairs of color skin images and their corresponding vein visualization results.

Unlike spider veins, feeder veins are often beneath the surface and invisible to naked eyes. If they can be visualized from color images, it will greatly benefit the following treatment. We applied the regression-based method to some spider vein images collected from the internet,^33^^–^^36^ and the results are shown in Fig. 11. In Fig. 11, we can see that the visualized veins join the surface spider vein clusters and are larger, which are in accord with the definition of feeder veins. Therefore, we believe that the proposed regression-based method can successfully visualize feeder veins from spider vein images and can assist in the treatment of spider veins.

## 5 Conclusion

We propose a vein visualization method from color images. The proposed method can achieve clear vein visualization results without any professional equipment. Compared with existing methods, this approach is more accurate and does not require huge amounts of training data. Based on the difference in optical properties between venous blood and generic tissue, we derive biophysical parameters from the spectral reflectance images reconstructed by Wiener estimation. MC simulation is adopted to get prior information. Vein patterns are visualized from the distribution of blood. The effect of illumination and body surface on skin color can be minimized through image preprocessing. Experimental results indicate that the proposed method can visualize veins clearly and correctly. It also shows that the method has the potential to provide the location of veins in the treatment of spider veins. This method performs on a per-pixel basis; in the future, we will consider utilizing the structures of veins and combining the neighboring pixels to improve the visualization results as well as to reduce noise.
